# Graphene-Enhanced FePO_4_ Composites with Superior Electrochemical Performance for Lithium-Ion Batteries

**DOI:** 10.3390/ma18153604

**Published:** 2025-07-31

**Authors:** Jinde Yu, Shuchun Hu, Yaohan Zhang, Yin Liu, Wenjuan Ren, Aipeng Zhu, Yanqi Feng, Zhe Wang, Dunan Rao, Yuqin Yang, Heng Zhang, Runhan Liu, Shunying Chang

**Affiliations:** 1 Yibin Research Institute, Chengdu Technological University, Chengdu 611730, China; kinder0905@163.com (J.Y.); liuyin9114@163.com (Y.L.); woshiwenjuanhehe@163.com (W.R.); zhuaipeng@cdtu.edu.cn (A.Z.); fyqsust@foxmail.com (Y.F.); wzhe3995@163.com (Z.W.); 2 School of Materials and Environmental Engineering, Chengdu Technological University, Chengdu 611730, China; 18008258129@163.com (Y.Y.); 15680614315@163.com (H.Z.); 15680733354@163.com (R.L.); 13076066737@163.com (S.C.); 3 School of Materials Science and Engineering, Southwest Jiaotong University, Chengdu 610031, China; zyaohan@163.com (Y.Z.); rdunan@163.com (D.R.)

**Keywords:** lithium-ion batteries, olivine-type FePO_4_, graphene composite

## Abstract

In this study, we successfully synthesized olivine-type FePO_4_ via an in situ oxidation method and further developed two composite cathode materials (o-FePO_4_-1/GR-1 and o-FePO_4_-1/GR-2) by incorporating graphene. The composites were characterized using scanning electron microscopy (SEM), X-ray diffraction (XRD), and X-ray Photoelectron Spectroscopy (XPS), revealing a three-dimensional porous layered structure with an enhanced surface area and strong interaction between FePO_4_ nanoparticles and graphene layers. Electrochemical tests demonstrated that the composite electrodes exhibited significantly improved performance compared to pristine FePO_4_, with discharge capacities of 147 mAh g^−1^ at 1C and 163 mAh g^−1^ at 0.1C for o-FePO_4_-1/GR-2, approaching the level of LiFePO_4_. The incorporation of graphene effectively enhanced the electrochemical reaction kinetics, highlighting the innovation of our method in developing high-performance cathode materials for lithium-ion batteries.

## 1. Introduction

Lithium-ion batteries (LIBs) have become a cornerstone in modern energy storage technology due to their high energy density, long cycle life, and high efficiency [1]. Among various cathode materials, LiFePO_4_ (LFP) has garnered significant attention due to its high theoretical specific capacity (170 mAh g^−1^), excellent thermal stability, and environmental benignity [2,3,4]. However, the low electronic conductivity and ionic diffusivity of LFP often limit its rate capability and rapid charge/discharge performance [5,6].

To address these limitations, researchers have explored various strategies to enhance the electrochemical performance of LFP [7,8]. One promising approach is the synthesis of composite materials by incorporating conductive additives [9] such as graphene. Graphene, with its high electrical conductivity, large surface area, and excellent mechanical flexibility, has been proven to be an effective material for improving the overall performance of LIBs [10,11]. For example, Hu et al. demonstrated that graphene-modified LFP cathodes can achieve performance beyond their theoretical capacity [12]. Similarly, Liu et al. showed that the addition of graphene nanosheets can significantly enhance the morphology and electrochemical performance of LFP particles [13]. Fathollahi et al. reported that LFP/graphene nanocomposites prepared by a one-step hydrothermal method exhibited improved electrochemical properties [14]. Tang et al. demonstrated that a carbon gel-assisted synthesis of C-LFP/graphene layers can achieve high rates and cycle performances [15]. These studies underscore the importance of graphene incorporation in enhancing the electrochemical performance of LFP-based materials. These studies highlight the potential of graphene-based composites in improving the performance and safety of LIBs [16,17,18].

In recent years, the development of novel composite materials based on FePO_4_ has also attracted considerable interest [19,20,21]. FePO_4_, an oxidation product of LFP, has been studied as a potential cathode material due to its similar crystal structure and theoretical capacity [22,23,24]. However, FePO_4_ also suffers from poor conductivity and slow lithium-ion diffusion, mainly due to the similar olivine-type structure as LFP, which restricts its practical application. Therefore, the incorporation of conductive material into FePO_4_ to form a composite material is expected to significantly enhance its electrochemical performance [25].

Moreover, previous studies on FePO_4_/graphene composites typically employed a two-step synthesis route, where FePO_4_ was first prepared separately and then physically mixed with graphene [25], which may often lead to inhomogeneous distribution of FePO_4_ particles and severe aggregation due to poor interfacial contact. In addition, conventional methods for crystallizing FePO_4_ rely on energy-intensive processes such as high-temperature calcination [21], which not only increase production costs but also risk damaging the graphene’s conductive network due to excessive defect formation and particle coarsening. Therefore, a simpler process and milder reaction conditions are required to prepare high-performance lithium-ion batteries based on FePO_4_.

In this study, we develop high-performance olivine-type FePO_4_/graphene composite cathodes through an innovative in situ oxidation approach, which directly converts LFP to FePO_4_ via a mild (NH_4_)_2_S_2_O_8_-mediated oxidation during composite formation. This one-pot process relatively ensures uniform dispersion of FePO_4_ nanoparticles on graphene while minimizing particle aggregation. The resulting composite materials, designated as o-FePO_4_-1/GR-1 and o-FePO_4_-1/GR-2, were characterized using various techniques such as scanning electron microscopy (SEM), X-ray diffraction (XRD), and X-ray Photoelectron Spectroscopy (XPS). The electrochemical performance of these composites as cathode materials in LIBs was evaluated in terms of cyclic voltammetry (CV), charge/discharge capacity, cycling stability, and rate capability. Our strategy addresses the intrinsic limitations of FePO_4_ materials by constructing three-dimensional layered conductive networks, achieving electrochemical performance competitive with conventional LFP cathodes. This graphene-enhanced architecture not only overcomes the traditional conductivity constraints of FePO_4_ systems but also establishes a new paradigm for designing lithium-free cathode materials without compromising performance stability, which provides a new perspective on the development of high-performance cathode materials for LIBs based on FePO_4_.

## 2. Materials and Methods

The synthesis of olivine-type FePO_4_ required the following reagents: ammonium persulfate ((NH_4_)_2_S_2_O_8_, AR, Chengdu, China), LFP (Battery grade, China), hydrogen peroxide (H_2_O_2_, 30 wt.%), graphene (Battery grade, Neware Technology Co., Ltd., Shenzhen, China), and deionized water. For electrode preparation, polyvinylidene fluoride (PVDF, 99.99%), Super P Li carbon black (Battery grade, Neware Technology Co., Ltd., China), N-methyl-2-pyrrolidone (NMP, AR), and aluminum foil (Battery grade, Neware Technology Co., Ltd., China) were used. Battery assembly components included the following: 14 mm diameter lithium metal anodes, 16 mm polypropylene separators, spacers, springs, and 1 mol/L lithium hexafluorophosphate (LiPF6, Battery grade, Neware Technology Co., Ltd., China) electrolyte solution.

A 0.1 M (NH_4_)_2_S_2_O_8_ aqueous solution (100 mL) was prepared by dissolving 2.28 g of (NH_4_)_2_S_2_O_8_. For o-FePO_4_-1 synthesis, 0.64 g of LFP powder was added to 20 mL of the (NH_4_)_2_S_2_O_8_ solution (molar ratio 1:2) and stirred at 25 °C for 24 h. After filtration and drying, 0.6 g of black FePO_4_ powder (denoted o-FePO_4_-1) was obtained [26]. o-FePO_4_-2, o-FePO_4_-3, and o-FePO_4_-4 were prepared similarly but with different (NH_4_)_2_S_2_O_8_:LFP molar ratios (0.75:2, 0.5:2, and 0.25:2, respectively), corresponding to 15 mL, 10 mL, and 5 mL of 0.1 M (NH_4_)_2_S_2_O_8_ solution.

For o-FePO_4_-1/GR-1 (GR as the abbreviation of graphene): 20 mL (0.1 M) (NH_4_)_2_S_2_O_8_ solution was mixed with 0.064 g of graphene and stirred at 25 °C for 1 h, followed by addition of 0.64 g LFP and continued stirring for 23 h. For o-FePO_4_-1/GR-2, LFP was first mixed with (NH_4_)_2_S_2_O_8_ solution for 1 h before graphene addition. Both composites contained 10 wt.% graphene.

The active materials (o-FePO_4_ or its composites), Super P Li, and PVDF were mixed in NMP at an 8:1:1 mass ratio (0.4 g:0.05 g:0.05 g) for 24 h. The slurry was coated onto Al foil, dried at 100 °C for 1 h, and then vacuum-dried at 120 °C for 12 h. Electrode disks (14 mm in diameter) with active material loading of 2.0–2.5 mg/cm^2^ were prepared.

The CR2025 coin cells were assembled in an Ar-filled glovebox (<1 ppm H_2_O/O_2_) by sequentially stacking: negative can, electrolyte (2–3 drops), working electrode, separator, additional electrolyte (5–7 drops), Li counter electrode, spacers, spring, and positive can. Cells were sealed under pressure and rested for 12 h before testing.

Detailed material characterization and electrochemical measurements are provided in the Supplementary Information. All electrochemical performance data were obtained from at least four parallel coin cells for each sample, and the reported results represent averaged values.

## 3. Results

**Figure 1:**
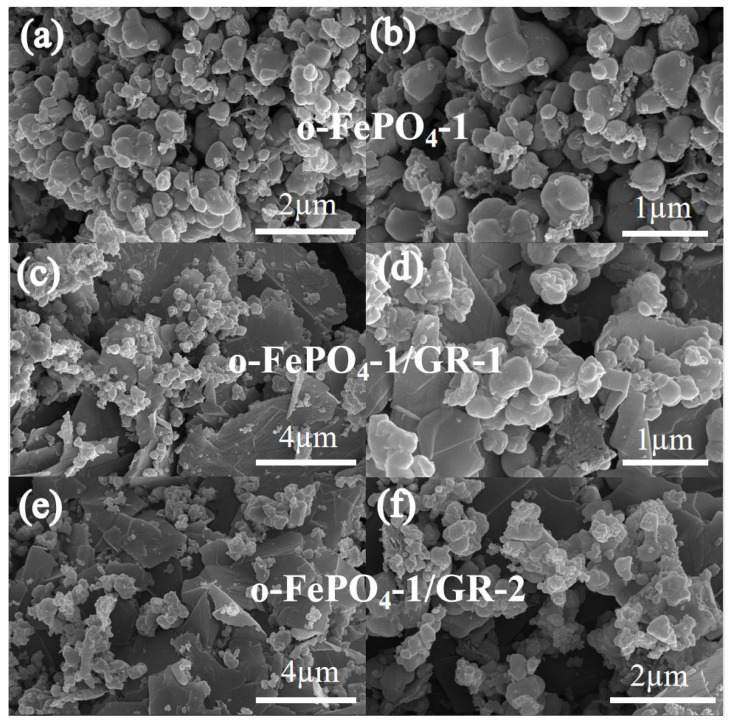
The SEM images and corresponding magnified views of o-FePO_4_-1 and o-FePO_4_-1/GR composites: (**a**,**b**) o-FePO_4_-1; (**c**,**d**) o-FePO_4_-1/GR-1; (**e**,**f**) o-FePO_4_-1/GR-2.

Figure 1 depicts SEM images of o-FePO_4_-1, o-FePO_4_-1/GR-1, and o-FePO_4_-1/GR-2 products. From the images, it is evident that the o-FePO_4_-1/GR products, o-FePO_4_-1/GR-1 and o-FePO_4_-1/GR-2, exhibit a three-dimensional porous layered composite structure. Compared to o-FePO_4_-1 nanoparticles, the nanoparticle sizes of both composites have decreased to between 50 and 100 nm. This reduction is attributed to the formation of a graphene two-dimensional layered structure within the composite material, which provides a high surface area. This layered structure facilitates the dispersion of o-FePO_4_ nanoparticles on the graphene surface, limiting the aggregation of o-FePO_4_ particles and thereby reducing their size within the composite. SEM results indicate that o-FePO_4_ nanoparticles are distributed on graphene sheets, with a strong interaction between the o-FePO_4_ nanoparticles and graphene layers, forming the o-FePO_4_-1/GR composite products. Moreover, from Figure S1, it can be observed that o-FePO_4_-1, o-FePO_4_-2, o-FePO_4_-3, and o-FePO_4_-4 all exhibit porous spherical structures. The particles of these four o-FePO_4_ products are exactly the same in shape and size, with the particle diameters ranging from 100 to 200 nm, a result governed by the principle of minimal free energy. It also indicates that the dosage of the oxidant basically has no effect on the size of the particles.

**Figure 2:**
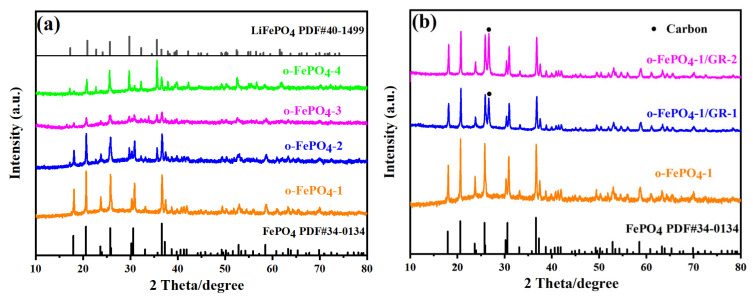
XRD patterns of o-FePO_4_ and o-FePO_4_-1/GR composites prepared under different conditions:. (**a**) o-FePO_4_-1, o-FePO_4_-2, o-FePO_4_-3, o-FePO_4_-4; (**b**) o-FePO_4_-1/GR-1 and o-FePO_4_-1/GR-2.

Figure 2a shows the XRD patterns of four types of o-FePO_4_ products. The pattern of o-FePO_4_-1 perfectly matches the standard card#34-0134 for olivine-type FePO_4_, with its characteristic peak located at 36.6°. As the amount of oxidant (NH_4_)_2_S_2_O_8_ gradually decreases from o-FePO_4_-1 to o-FePO_4_-4, the intensity of the characteristic peak at 36.6° visibly weakens in the XRD patterns of these four products, while the intensity of the peak at 29.68° strengthens. Notably, the peak at 29.68° corresponds to LFP, indicating that the reduced (NH_4_)_2_S_2_O_8_ dosage leads to progressively weaker oxidation of LFP. Consequently, the FePO_4_ content in these products gradually decreases, while the LFP content increases, ultimately resulting in the XRD pattern of o-FePO_4_-4 more closely resembling the standard card#40-1499 for LFP. Additionally, Figure 2b presents the comparative XRD patterns of two composites, o-FePO_4_-1/GR-1 and o-FePO_4_-1/GR-2, alongside o-FePO_4_-1. All three patterns align well with the peaks of standard card#34-0134. Furthermore, the XRD patterns of both composites in Figure 2b exhibit a characteristic carbon peak (marked with black dots at 2θ = 26.6°), which is absent in the pattern of o-FePO_4_-1. This confirms the successful preparation of the two composites, o-FePO_4_-1/GR-1 and o-FePO_4_-1/GR-2, and demonstrates that the olivine-type structure of FePO_4_ remains unchanged during the composite formation process. Furthermore, the crystallite sizes of o-FePO_4_-1, o-FePO_4_-1/GR-1, and o-FePO_4_-1/GR-2 were calculated from XRD data using the Scherrer equation, yielding values of 52.4 nm, 41.3 nm, and 35.8 nm, respectively. This progressive reduction in crystallite size correlates well with the SEM observations (Figure 1). The discrepancy between crystallite size (XRD) and particle size (SEM) suggests that each observed particle in the composites consists of multiple crystallites, and graphene incorporation effectively reduces both primary particle size and crystallite dimensions during synthesis. Obviously, o-FePO_4_-1/GR-2 shows the smallest crystallite size, consistent with its superior electrochemical performance, as the increased grain boundaries may enhance reaction kinetics.

**Figure 3:**
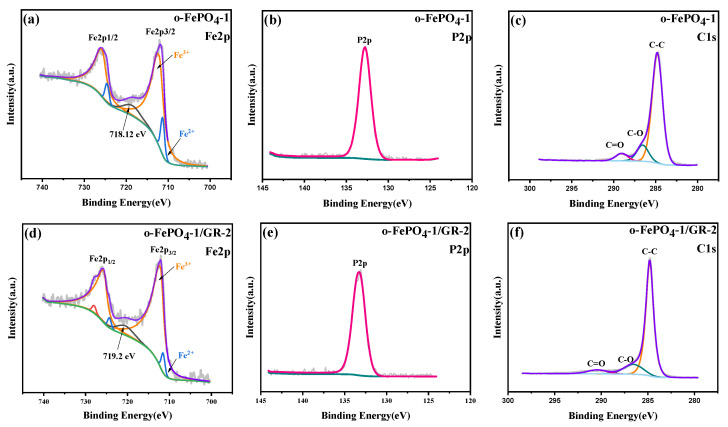
XPS spectra of the o-FePO_4_-1 and o-FePO_4_-1/GR-2 composite prepared under different conditions: (**a**–**c**) o-FePO_4_-1; (**d**–**f**) o-FePO_4_-1/GR-2.

XPS can provide insights into the elemental composition, atomic valence states, molecular structure, and surface chemistry of materials. Figure S2 displays the XPS survey spectra of four o-FePO_4_ samples and two o-FePO_4_-1/GR composites, confirming the presence of C, P, O, and Fe in all samples. High-resolution XPS spectra were further obtained for the Fe, P, and C regions of the two composites. As shown in Figure 3a,d, rigorous peak fitting of the Fe 2p spectra reveals the presence of Fe^3+^ (712.15 eV) and Fe^2+^ (711.3 eV), with quantitative analysis showing Fe^3+^/Fe^2+^ ratios of 92:8 for o-FePO_4_-1 and 96:4 for o-FePO_4_-1/GR-2, confirming the dominant Fe^3+^ content in both materials. The actual Fe^2+^ content may be even lower than these values due to the potential X-ray-induced reduction during measurements, as suggested by the presence of characteristic shake-up satellite peaks at around 718.5 eV that serve as unambiguous Fe^3+^ fingerprints [20,26]. This conclusion is also supported by the absence of LFP diffraction peaks at 29.68° in XRD patterns, which corroborates the almost complete oxidation of the precursor material. In the P 2p region (Figure 3b,e), a distinct single peak at 133.8 eV confirms the presence of PO_4_^3−^ groups. Additionally, the C 1s spectra (Figure 3c,f) exhibit a dominant peak at 284.8 eV, corresponding to sp^2^ hybridized carbon (C=C/C–C), confirming the preservation of graphene’s conductive backbone. However, upon detailed spectral deconvolution, minor peaks at 286.3 eV (C–O) and 288.5 eV (C=O) were identified [24], indicating the presence of trace oxygen-containing functional groups. This may be attributed to the partial oxidation of graphene edges by sulfate radicals generated during the in situ synthesis [27]. The low intensity of these peaks suggests limited defect-containing reduced graphene oxide introduction, consistent with edge-selectively functionalized graphene rather than heavily oxidized graphene oxide. For simplicity, the term “graphene” is used throughout this study to refer to the edge-selectively functionalized graphene in the composites. In summary, combined with SEM, XRD, and XPS characterizations, these results demonstrate the successful synthesis of o-FePO_4_ via an in situ oxidation method, as well as the preparation of two distinct o-FePO_4_-1/GR composites.

**Figure 4:**
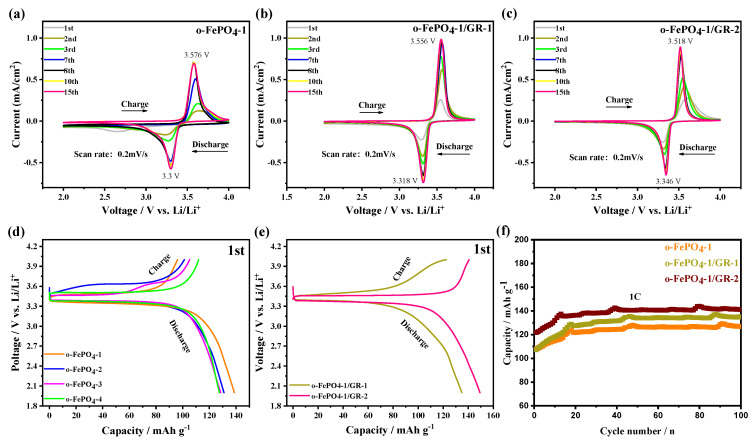
Electrochemical performance of the o-FePO_4_ and o-FePO_4_-1/GR composites: (**a**–**c**) CV profiles of the o-FePO_4_-1 and o-FePO_4_-1/GR composites electrodes. (**d**,**e**) Initial charge/discharge profiles of o-FePO_4_ and o-FePO_4_-1/GR composite electrodes at 0.1C rate. (**f**) Cyclic charge/discharge performance of o-FePO_4_-1, o-FePO_4_-1/GR-1, o-FePO_4_-1/GR-2 electrodes at 1C rate.

Figure 4a–c present the CV profiles of four o-FePO_4_ samples and two o-FePO_4_-1/GR composites. A distinct pair of redox peaks is observed around 3.5 V, which corresponds well with the Fe(III)/Fe(II) redox potential. However, the reproducibility of CV curves varies among different products. For o-FePO_4_-1 and o-FePO_4_-2 electrodes, good reproducibility is achieved after the eighth cycle, while o-FePO_4_-3 and o-FePO_4_-4 electrodes demonstrate stable reproducibility after just three and two cycles, respectively (Figure S3). Furthermore, it is found that the current for o-FePO_4_-1, o-FePO_4_-1/GR-1, and o-FePO_4_-1/GR-2 from the first cycle to the eighth cycle of the CV curves gradually increased and stabilized, which might be attributed to the wetting of the electrode/electrolyte interface and the initial formation of the solid electrolyte interphase. In addition, the ΔE_p_ values for o-FePO_4_-1, o-FePO_4_-1/GR-1, and o-FePO_4_-1/GR-2 in the 15th cycle were 0.28 V, 0.24 V, and 0.17 V, respectively, indicating that o-FePO_4_-1/GR-2 exhibits the enhanced reversibility of redox processes due to the rapid electron and ionic channels constructed by graphene incorporation, which can also be mutually verified by the subsequent cycle, rate performance, and electrochemical impedance spectroscopy (EIS) results.

The four o-FePO_4_ products and two o-FePO_4_-1/GR composites were employed as cathode-active materials in lithium-ion batteries, and their initial charge/discharge behaviors were evaluated at 0.1C (Figure 4d,e). The results reveal that all six electrodes exhibit flat voltage plateaus around 3.5 V during charge/discharge, consistent with the CV measurements. The initial Coulombic efficiencies of o-FePO_4_-1, o-FePO_4_-2, o-FePO_4_-3, and o-FePO_4_-4 electrodes were gradually increasing. Notably, the initial discharge capacities progressively decreased from o-FePO_4_-1 to o-FePO_4_-4. In contrast, the o-FePO_4_-1/GR-1 and o-FePO_4_-1/GR-2 composite electrodes demonstrated higher initial efficiencies than that of pure o-FePO_4_-1, with corresponding discharge capacities of 140 mAh g^−1^ and 150 mAh g^−1^. These initial charge/discharge tests clearly indicate that the composite electrodes derived from o-FePO^4^-1 and GR exhibit superior performance in both discharge efficiency and capacity compared to the pristine o-FePO_4_-1 electrode.

Figure 4f presents the charge/discharge cycling profiles of o-FePO_4_-1, o-FePO_4_-1/GR-1, o-FePO_4_-1/GR-2, and LFP (Figure S4) electrodes at 1C rate. The results demonstrate that, after 100 cycles, the o-FePO_4_-1/GR-1, o-FePO_4_-1/GR-2, and LFP electrodes maintain discharge specific capacities of 135 mAh g^−1^, 140 mAh g^−1^, and 133 mAh g^−1^, respectively. The composite electrodes exhibited significantly higher specific capacities compared to the o-FePO_4_-1 electrode (126 mAh g^−1^). This enhancement can be attributed to the incorporation of graphene with high carrier mobility into o-FePO_4_-1, which substantially improved the electrochemical performance. Notably, the o-FePO_4_-1/GR-2 electrode displayed superior capacity retention relative to o-FePO_4_-1/GR-1, indicating better lithium storage capability, with its performance even exceeding that of the LFP electrode. In contrast, the o-FePO_4_-2, o-FePO_4_-3, and o-FePO_4_-4 electrodes delivered lower discharge specific capacities of 117 mAh g^−1^, 113 mAh g^−1^, and 111 mAh g^−1^ after 100 cycles at 1C, all substantially inferior to the composite electrodes. These results highlight the critical role of graphene in enhancing the cycling performance of o-FePO_4_-based cathode materials.

**Figure 5:**
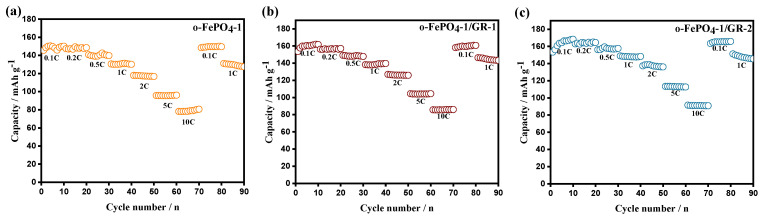
Rate capability curves of o-FePO_4_-1 (**a**), o-FePO_4_-1/GR-1 (**b**), and o-FePO_4_-1/GR-2 (**c**) electrodes at various rates from 0.1C to 10C.

Figure 5 displays the rate capability curves of o-FePO_4_-1, o-FePO_4_-1/GR-1, and o-FePO_4_-1/GR-2 electrodes tested from 0.1C to 10C. The results reveal that the discharge-specific capacities of all three electrodes gradually decrease with increasing current rates. While the capacity fading is relatively moderate within the 0.1–2C range, significant capacity reduction occurs at higher rates of 5C and 10C. The o-FePO_4_-1 electrode delivers discharge specific capacities of 160, 153, 140, 126, 120, 100, and 80 mAh g^−1^ at 0.1, 0.2, 0.5, 1, 2, 5, and 10C rates, respectively, consistently outperforming o-FePO_4_-2, o-FePO_4_-3, and o-FePO_4_-4 electrodes across all tested rates. The graphene-modified composites demonstrate further enhanced rate performance: o-FePO_4_-1/GR-1 exhibits capacities of 161, 156, 148, 135, 126, 104, and 85 mAh g^−1^, while o-FePO_4_-1/GR-2 shows 162, 160, 156, 140, 130, 105, and 87 mAh g^−1^ at corresponding rates. To contextualize the performance of our o-FePO_4_-1/GR composites, Table S1 compares their electrochemical properties with state-of-the-art LFP and FePO_4_-contained cathodes. Our composite materials (o-FePO_4_-1/GR-2) demonstrate competitive performance, achieving comparable discharge capacity and cycling stability to LFP-based cathodes. Notably, o-FePO_4_-1/GR-2 surpasses most FePO_4_-based materials [23,24] in capacity retention and discharge capacity, highlighting the efficacy of our graphene incorporation strategy.

**Figure 6:**
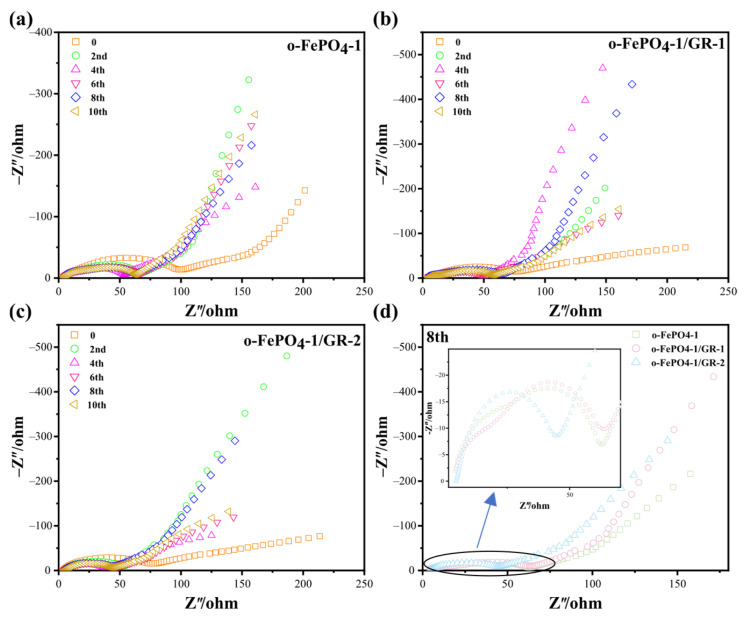
EIS plots of (**a**) o-FePO_4_-1, (**b**) o-FePO_4_-1/GR-1, and (**c**) o-FePO_4_-1/GR-2. (**d**) Comparison of the 8th circle diagrams of all three materials.

EIS is a common technique that characterizes electrode/electrolyte interfaces by measuring the frequency-dependent impedance to reveal charge transfer kinetics, interfacial stability, and mass transport properties in electrochemical systems [28,29]. Therefore, we systematically characterized the impedance properties of o-FePO_4_-1, o-FePO_4_-1/GR-1, and o-FePO_4_-1/GR-2 with 10 consecutive cycling measurements to comprehensively evaluate their interfacial evolution and stability during electrochemical operation. The EIS dates of all three materials (Figure 6) exhibit similar features consisting of a semicircle in the high-to-medium frequency region and a linear Warburg tail in the low-frequency region, corresponding to charge transfer and Li^+^ diffusion processes, respectively. The rapid stabilization of all electrodes after the second cycle suggests that all three materials achieve stable electrode/electrolyte interfaces more quickly. In order to more accurately compare the impedance characteristics of these different materials, we selected the eighth circle impedance diagrams of the three materials for comparison, shown in Figure 6d. Analysis of the 8th cycle data reveals distinct differences in electrochemical behavior. The o-FePO_4_-1/GR-2 presents the smallest semicircle diameter and steepest Warburg slope, indicating superior charge transfer kinetics and Li^+^ diffusion efficiency. These favorable impedance characteristics directly correlate with its highest electrochemical performance (163 mAh g^−1^ at 0.1C). The intermediate performance of o-FePO_4_-1/GR-1 aligns with its moderately higher charge transfer resistance and similar Warburg slope to that of o-FePO_4_-1/GR-2. While the graphene incorporation improves ion transport compared to pristine o-FePO_4_-1, the less optimal architecture results in greater interfacial resistance. Notably, pristine o-FePO_4_-1 shows the largest semicircle diameter and shallowest Warburg slope, explaining its relatively poor performance due to both sluggish charge transfer and limited Li^+^ mobility. The impedance parameters confirm that o-FePO_4_-1/GR-2′s optimal graphene distribution creates the most efficient conductive network, enabling simultaneous enhancement of both electronic and ionic transport.

## 4. Conclusions

In summary, this study successfully synthesized olivine-type FePO_4_ through an in situ oxidation method and developed two graphene-incorporated composite cathode materials (o-FePO_4_-1/GR-1 and o-FePO_4_-1/GR-2). The composites exhibited significantly enhanced electrochemical performance compared to pristine FePO_4_. Specifically, the o-FePO_4_-1/GR-2 composite demonstrated a discharge capacity of 147 mAh g^−1^ at 1C and 163 mAh g^−1^ at 0.1C, approaching the performance of LFP. These improvements were primarily attributed to the high carrier mobility of graphene, which facilitated charge transfer and improved the overall electrochemical reaction kinetics within the composite electrodes. This work highlights the effectiveness of the in situ oxidation method and graphene incorporation in developing high-performance cathode materials for lithium-ion batteries, providing a promising approach for future energy storage applications.

## Data Availability

The original contributions presented in this study are included in the article/Supplementary Material. Further inquiries can be directed to the corresponding author.

## Supplementary Materials

The following supporting information can be downloaded at https://www.mdpi.com/article/10.3390/ma18153604/s1, Figure S1: The SEM images and corresponding magnified views of o-FePO_4_: (a,b) o-FePO_4_-1; (c,d) o-FePO_4_-2; (e,f) o-FePO_4_-3; (g,h) o-FePO_4_-4; Figure S2: XPS survey of the o-FePO_4_ and o-FePO_4_-1/GR composites prepared under different conditions: (a) o-FePO_4_-1; (b) o-FePO_4_-2. (c) o-FePO_4_-3; (d) o-FePO_4_-4; (e) o-FePO_4_-1/GR-1; (f) o-FePO_4_-1/GR-2; Figure S3: CV profiles of the o-FePO_4_-2, o-FePO_4_-3 and o-FePO_4_-4 electrodes; Figure S4: Cyclic charge/discharge performance of o-FePO_4_-2, o-FePO_4_-3, o-FePO_4_-4, and LiFePO_4_ electrodes at 1C rate; and Figure S5: Rate capability curves of o-FePO_4_-2, o-FePO_4_-3 and o-FePO_4_-4 electrodes at various rates from 0.1C to 10C. Table S1: Comparative electrochemical performance of LFP and FePO_4_-contained cathode materials for LIBs. The citation numbers of the references in the table correspond one-to-one with those in the main text.

## Abbreviations

The following abbreviations are used in this manuscript:

SEM	Scanning Electron Microscopy
XRD	X-ray Diffraction
XPS	X-ray Photoelectron Spectroscopy
LFP	LiFePO_4_
LIBs	Lithium-Ion Batteries
o-FePO_4_	Olivine-Type FePO_4_
