# Supplementary material for: Graphene-Enhanced FePO4 Composites with Superior Electrochemical Performance for Lithium-Ion Batteries
Source: Materials (Basel). 2025 Jul 31;18(15):3604. doi: 10.3390/ma18153604 (PMC12348126; doi:10.3390/ma18153604)
Supplement: Supplementary file 1 [file materials-18-03604-s001.zip › materials-3739589-supplementary.pdf]

Graphene-Enhanced $FePO_4$ Composites with Superior Electrochemical Performance for Lithium-Ion Batteries

Jinde Yu [1], Shuchun Hu [1,2,*], Yaohan Zhang [3], Yin Liu [1,2], Wenjuan Ren [1], Aipeng Zhu [1], Yanqi Feng [1,2], Zhe Wang [1,2], Dunan Rao [3], Yuqin Yang [2], Heng Zhang [2], Runhan Liu [2] and Shunying Chang [2]

[1] Yibin Research Institute, Chengdu Technological University, Chengdu 611730, China;
kinder0905@163.com (J.Y.); liuyin9114@163.com (Y.L.);
woshiwenjuanhehe@163.com (W.R.);
zhuaipeng@cdtu.edu.cn (A.Z.); fyqsust@foxmail.com (Y.F.);
wzhe3995@163.com (Z.W.)

[2] School of Materials and Environmental Engineering, Chengdu Technological University,
Chengdu 611730, China; 18008258129@163.com (Y.Y.); 15680614315@163.com (H.Z.);
15680733354@163.com (R.L.); 13076066737@163.com (S.C.)

[3] School of Materials Science and Engineering, Southwest Jiaotong University, Chengdu 610031, China;
zyaohan@163.com (Y.Z.); rdunan@163.com (D.R.)

* Correspondence: schu@home.swjtu.edu.cn

Materials Characterization and Electrochemical Measurements

Microstructural characterization of olivine-type o-$FePO_4$ and its composites was performed using a Thermo Scientific Apreo 2C scanning electron microscope (Thermo Fisher Scientific, USA) operated at 20 kV accelerating voltage.

X-ray diffraction patterns were collected on a Rigaku Ultima IV multipurpose X-ray diffractometer (Rigaku Corporation, Japan) with Cu-Kα radiation (λ = 1.5406 Å) at 40 kV and 30 mA, scanning from 10° to 80° (2θ) with a step size of 0.02°.

X-ray photoelectron spectroscopy measurements were conducted using a Thermo Scientific K-Alpha spectrometer (Thermo Fisher Scientific, USA) equipped with an Al-Kα X-ray source (1486.6 eV) operated at 12 kV and 6 mA. High-resolution spectra were collected for elemental composition and valence state analysis.

Electrochemical performance (Cyclic Voltammetry) was evaluated using a CT-4000T-5V6A battery testing system within a voltage window of 2.0-4.0 V vs. Li^+/Li at room temperature. Specific capacities were calculated based on the active material mass of o-$FePO_4$ products. The geometric area of the working electrode was measured with a micrometer to be 1.54 cm^2.

Electrochemical measurements (Charge/Discharge Testing) were performed on a CHI-7660E electrochemical workstation using a three-electrode configuration: o-$FePO_4$-based composites as working electrode, lithium foil as both counter and reference electrodes. CV scans were conducted between 2.0-4.0 V at a scan rate of 0.2 mV/s, initiating from high to low potential.

Electrochemical Impedance Spectroscopy measurements were carried out on the CHI-7660E system across a frequency range of 0.01 Hz to 1 MHz with an AC amplitude of 0 mV.

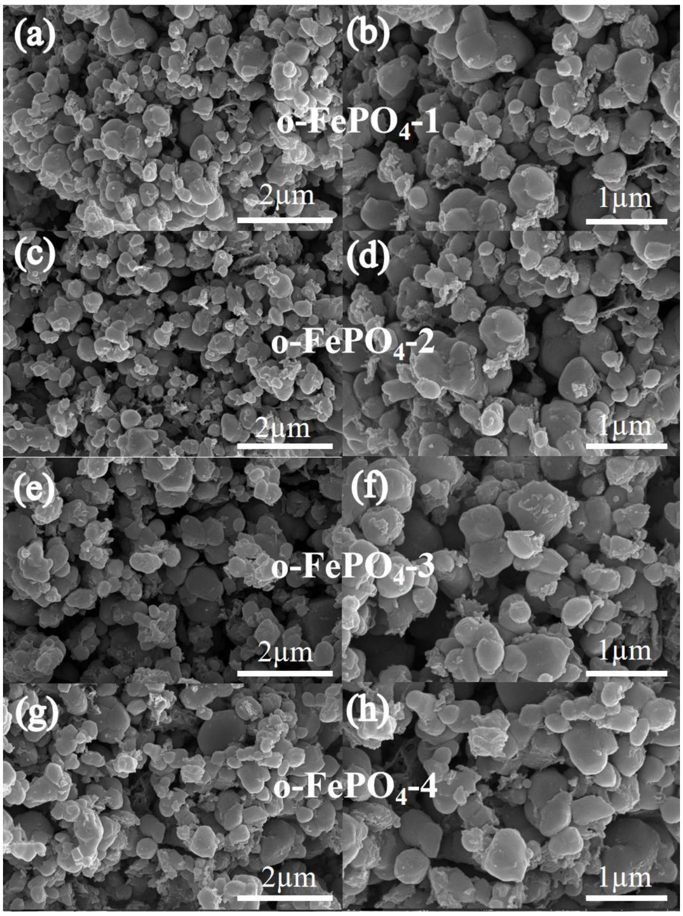

Figure S1 The SEM images and corresponding magnified views of o-$FePO_4$: (a-b) o-$FePO_4$-1; (c-d) o-$FePO_4$-2; (e-f) o-$FePO_4$-3; (g-h) o-$FePO_4$-4.

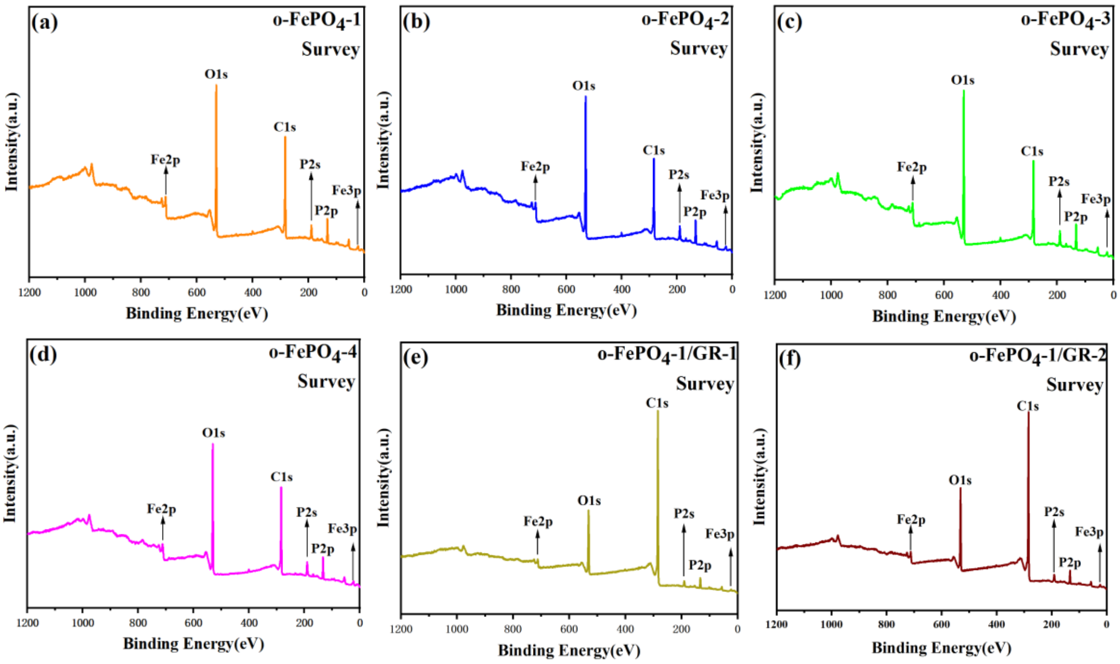

Figure S2 XPS survey of the o-$FePO_4$ and o-$FePO_4$-1/GR composites prepared under different conditions: (a) o-$FePO_4$-1; (b) o-$FePO_4$-2. (c) o-$FePO_4$-3; (d) o-$FePO_4$-4; (e) o-$FePO_4$-1/GR-1; (f) o-$FePO_4$-1/GR-2.

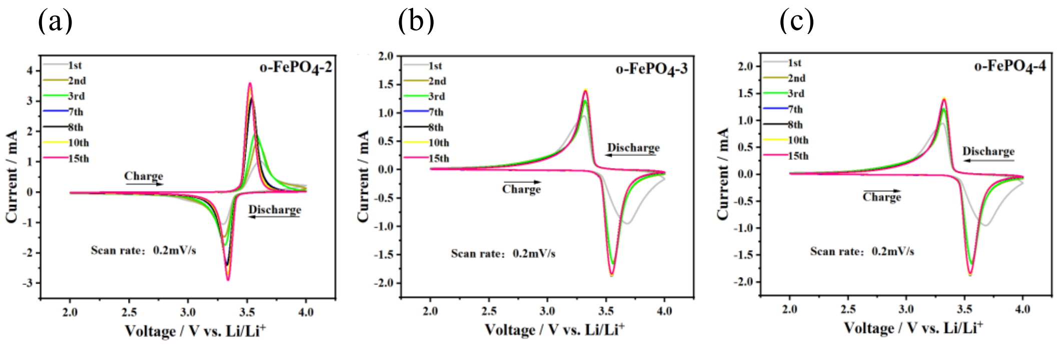

Figure S3 CV profiles of the o-$FePO_4$-2、o-$FePO_4$-3 and o-$FePO_4$-4 electrodes.

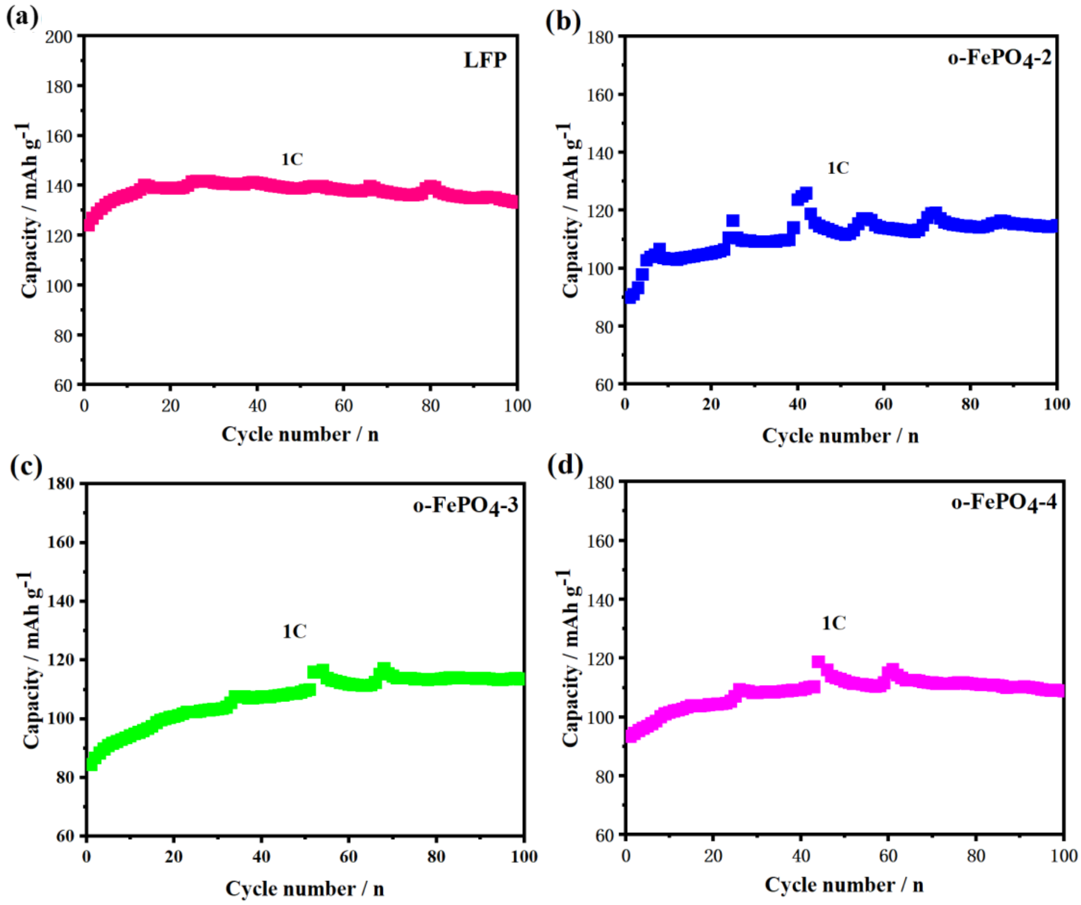

Figure S4 Cyclic charge/discharge performance of o-$FePO_4$-2, o-$FePO_4$-3, o-$FePO_4$, and $LiFePO_4$ electrodes at 1C rate.

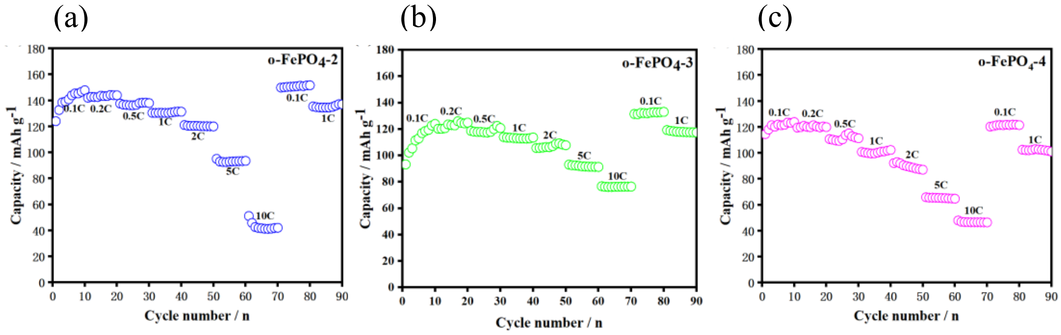

Figure S5 Rate capability curves of o-$FePO_4$-2, o-$FePO_4$-3 and o-$FePO_4$-4 electrodes at various rates from 0.1 C to 10 C.

Table S1. Comparative electrochemical performance of LFP and $FePO_4$-contained cathode materials for LIBs.

Material	Discharge Capacity ($mAh\ g^{-1}$)	Rate Performance (Capacity at 10C)	Cycle Stability (Capacity Retention after 100 cycles)	Reference
o-$FePO_4$-1/GR-2 (This work)	163 (0.1C), 147 (1C)	87 $mAh\ g^{-1}$	100% (1C)	-
LFP/graphene (3D porous)	155 (0.2C), 132 (5C)	92 $mAh\ g^{-1}$	98% (1C)	[17]
LFP/graphene (cLFP)	160 (0.1C), 138 (1.3C)	93 $mAh\ g^{-1}$	97% (1.3C)	[12]
LFP/N-rGO	160 (0.2C), 140 (1C)	86 $mAh\ g^{-1}$	96% (0.2C)	[6]
LFP/graphene (microwave)	162 (0.5C), 162 (1C)	132 $mAh\ g^{-1}$ (16C)	-	[16]
$FePO_4$/GO	120 (0.1C), 100 (1C)	55 $mAh\ g^{-1}$	83% (1C)	[24]
$FePO_4$/MWCNTs	160 (0.1C), 140 (1C)	110 $mAh\ g^{-1}$	100% (1C)	[19]
R-FePO4	151 (0.1C), 134 (1C)	-	93% (1C)	[23]

*The citation numbers of the references in the table correspond one-to-one with those in the main text.
